# A Sense of Continuity in Mortality? Exploring Science-Oriented Finns’ Views on Afterdeath

**DOI:** 10.1177/00302228211038820

**Published:** 2021-08-18

**Authors:** Roosa Haimila, Elisa Muraja

**Affiliations:** 1 Study of Religion, Department of Cultures, Faculty of Arts / Doctoral Programme in Theology and Religious Studies, Faculty of Theology, University of Helsinki, Finland; 2 Faculty of Theology, University of Helsinki, Finland

**Keywords:** death, afterlife, science and religion, atheism, Finland

## Abstract

Endorsement of science might entail a belief in “secular death”, in which an individual faces annihilation as the bodily functions cease. In this article, we examine what science-oriented individuals think happens to humans after death. Does endorsement of science entail views on human annihilation or do people also express continuity beliefs? The open-ended responses of 387 Finns were analysed. The respondents were recruited online via organisations that promote science and research. The results suggest that while science-oriented Finns mainly endorsed annihilation and secular death, some also expressed (mostly nonreligious) views on continuation, e.g., in social bonds and nature. Secular forms of continuity were more likely mentioned by unbelievers, while theist respondents relied primarily on afterlife beliefs.

Scholars have long suggested that a religious afterlife belief is comforting for humans (Benore & Park, 2004; Vail et al., 2010). Similarly, some might suggest that science offers a bleak and gloomy reality especially when we consider what happens when people die. Some scholars have associated scientific knowledge with a secular conception of death, in which the individual annihilates as the bodily functions cease (Harris & Giménez, 2005; for discussion, see Hodge, 2016, 2018). Consonantly, individuals who hold science in high regard might perceive death as “the end” and not endorse continuity after dying (Carroll, 2016, p. 216; Dawkins, 2006). However, several issues cast doubt on this view. Although science and religious belief are often portrayed as conflicting, studies have found that people in various cultures utilise both religious and scientific explanations to make sense of death (Astuti & Harris, 2008; Harris & Giménez, 2005; Legare & Shtulman, 2018; Watson-Jones et al., 2017).^1^ Prior literature also suggests that humans may be prone to supernatural continuity beliefs (Bering, 2002; Hodge, 2016). Therefore, people might hold both supernatural and scientific views on death also in contexts that are often perceived as secular (cf. Bering et al., 2005).^2^

Furthermore, although beliefs on continuity after death *might be* supernatural, it is unclear whether this is necessarily so. According to the belief replacement hypothesis, secular beliefs can serve psychological functions that have been associated with religion (Farias, 2013). If humans are inclined to perceive continuity after death (Darrell & Pyszczynski, 2016; Hodge, 2016), they may also hold naturalistic views on continuity that operate with secular terms. What might these be? Some have suggested that people often exceed the finality of death with religious afterlife belief or by attaining *symbolic* immortality, e.g., through one’s children or legacy, or as a part of eternal nature (Darrell & Pyszczynski, 2016; Dechesne et al., 2003; Lifton, 1973; Vail et al., 2010).^3^ Still, we know little on whether people have integrated symbolic ways of continuation in their afterdeath imagery. Research on what people think happens after death has focused on the development and diversity of supernatural belief (Bibby, 2017; Lester et al., 2002; cf. also Astuti & Harris, 2008; Watson-Jones et al., 2017). Therefore, views that do not entail continuity of the consciousness have often been classified to a single category, such as “annihilation” (Burris & Bailey, 2009, p. 175) or “extinctionist” (Walker, 2000, p. 15; Singleton, 2012; cf. also Bering, 2002; Thalbourne, 1996). However, there are some data-driven studies that demonstrate the possible versatility of secular death beliefs (Jonsson & Aronsson, 2015; Toscani et al., 2003). For instance, Toscani et al. (2003) found that for individuals who lacked afterlife belief, the loss of individuality upon death did not only signify annihilation–some viewed it as merging oneself with a universal soul or as a return to the biological cycle (see also Manning, 2018).^4^

To summarise, people who hold science in high regard might be expected to endorse the scientific account of death, which some suggest entails the end of existence as the bodily functions cease. However, also science-oriented individuals’ reasoning about death might include a variety of views on annihilation and continuity, including religious afterlife belief. To date, there has been a dearth of studies on afterdeath beliefs of individuals who identify with research (cf. Walker, 2000).

In the present study, we explore the views of science-oriented Finns on death and human (in)finity. More precisely, we ask

- How do science-oriented individuals describe what happens to people after death?
- Does identification with science entail views on human annihilation, or do people also express a belief in continuity after death? If so, what continuity beliefs do the respondents hold?

The study aims to increase our knowledge on death beliefs among individuals who hold science in high regard.^5^ Moreover, relatively few have investigated current secular and religious death imagery in the Nordic context (however, see Arnett & Jensen, 2015; Jonsson & Aronsson, 2015; cf. Butters, 2021). The approach of the study is data-driven, although some of the initial categories applied in the analysis were based on previous literature and studies (van Mulukom et al., n.d.).

## Method and Participants

The data were collected with an online questionnaire, implemented on the GDPR-compliant LimeSurvey platform. We recruited the respondents via Finnish pro-research organisations.^6^ The invitation was first sent via email to research institutions and other research-affiliated organisations, followed by social media recruiting in Twitter, Facebook and selected discussion boards (for additional information, see Supplementary Material A). As an incentive, the respondents could participate in a raffle for an Amazon gift card (60€) and request a report on the results. Altogether, 683 respondents answered the questionnaire. In the sample, we included 387 respondents who answered the control question correctly (see Supplementary Material B; Oppenheimer et al., 2009). The sample’s respondents were of multiple genders (202 women; 170 men; 15 other/I don’t want to say), and their median age was 31–40 years (range across groups: 18–30, 31–40, 41–50, 51–65, and over 65 years). The respondents were highly educated (years of education *M* = 19.6, *SD* = 4.6), and half of them had worked in research institutions (50%; natural sciences 24%; humanities 14%; social sciences 9% and other research fields 4%). Most were not affiliated with any religious community (70%) and did not believe in God (70%). A minority of respondents were unsure of their God belief (17%) or reported that they believe in God (14%) (see also Haimila, 2020).

### Procedure

The respondents first answered open-ended questions on human origins, suffering and death. After the open-ended questions, the respondents completed scales measuring the significance of science, followed by items on religious and nonreligious cultural beliefs (see Haimila, 2020), the control question and demographic questions. In this article, we explore the respondents’ answers to the following open-ended questions:

- What do you think happens to us (humans) after death?
- Do you think the existence of an individual is finite or infinite? (Could you tell us why you think this is so?)^7^

For the analysis, we formulated a coding template around the central themes of annihilation and continuity after death. The initial categories were based on the pilot study, prior literature and observations from the first author’s previous study (van Mulukom et al., n.d.). However, we aimed for a data-driven analysis and added categories during the first coding rounds based on recurring themes in the responses. The template was formed and revised throughout three rounds of coding. During these, both authors independently coded the same randomly selected segments of data, after which the interrater reliability was explored, and revisions were made to the template based on mutual discussions. The internal consistency of the coding was good (96% percent agreement, Cohen’s kappa = .79, during the third round which contained 25% of the data). Finally, the first author coded the responses with the established coding template (Campbell et al., 2013; Syed & Nelson, 2015; see also, Legare & Gelman, 2008). The final coding template consisted of 21 categories. Next, we will describe establishing the coding template, starting with the formulation and content of the categories on annihilation and continuity.

### Annihilation—What Ceases to Exist

The responses on annihilation upon death often described it in a twofold manner: the body decays and the mind ceases to exist. We therefore established separate categories for cessation of bodily functions and the annihilation of the individual’s mind, conscious experience and thoughts. As an example, one respondent described what happens to humans after death by stating that “[t]he body is burnt or it decomposes and at some point nutrients for new life form from it. The mind ceases to exist. […]” (P2105).

However, many respondents described annihilation without a distinction between the mind and the body. Therefore, we added the category of *Annihilation no distinction*. In this category, we coded the responses that suggested that humans cease to exist upon death. For instance, one participant wrote: “We cease to be. We no longer exist after that [death]. […]” (P1942).

Some respondents made sense of annihilation by comparing it to experiences humans have during life. For instance, nonexistence after death was compared to deep sleep or unconsciousness that some respondents had experienced during anaesthesia. We coded these responses to the category *Annihilation life metaphor*.^8^

However, none of these categories entail that the respondent did not mention any kind of continuity after death. For instance, some participants stated that “nothing” happens after death (coded as Annihilation no distinction) and then went on to describe something that they do believe happens, e.g., that “we live on in the memories of others” (P1208).

### Continuity—What Remains

Based on the data and observations during the first author’s prior study, we established the category *Social continuity* to capture the responses that described continuation through social impact. This included subcategories for continuation in memories of close others (*Continuity close others*) or in other legacy (*Continuity societal*).^9^ We also included continuity in offspring, mainly based on earlier literature (Lifton, 1973; Vail et al., 2010). Due to prior findings on possibly intuitive mind–body dualism (Chudek et al., 2018), we expected that some respondents might mention belief in an afterlife of the mind or the soul. However, we wanted to mark these beliefs to separate categories, as we were interested in whether the science-oriented respondents would apply terminology that is at times associated to religiosity (*Continuity soul*) or more secular afterlife belief (*Continuity mind*).^10^ During the coding, we noticed that more categories were needed to capture the immortality beliefs. The category *Continuity religious/New Age* was established for continuity beliefs that did not refer to the soul but mentioned other terminology that might be perceived as religious in the Finnish context (e.g., Heaven) or regarded as spiritual and/or New Age belief (e.g., reincarnation). Due to recurrences in the data, we also created a category *Continuity same/new body* for an afterlife in the same body or a new animal or human body (e.g., reincarnation, resurrection body). As the first author expected the respondents might believe in circulation of nature based on a prior survey (van Mulukom et al., 2020), we established the category of Continuity in circulation of nature, later widening it to *Continuity natural laws*. Finally, we created a category for responses that expressed continuation after death but did not fit the criteria of the mentioned categories (*Continuity other*), e.g., when a respondent mentioned that after death we move on to “other airs” (P1263) or an unspecified place.

### Categories on Attitudes and the Role of Science

To further describe the responses, we created categories on attitudes that seemed to recur in the data. Therefore, we established a category for responses that mention continuity after death merely to reject the belief or otherwise criticise it (*Continuity rejection*). However, some people mentioned belief in continuity to express their conflicted feelings, e.g., if they were not sure whether or not to believe in an afterlife. These responses were categorised with the code *Conflicted*. In addition, we formulated codes for suggestions that belief in continuity is (or would be) comforting in face of mortality for the respondents themselves (*Comfort continuity*) and for responses that expressed something positive about mortality (*Death good/beneficial*). As we were interested in the possible role of science in annihilation and continuity beliefs, we also added the code *Science* for any mentions of science-related terminology (e.g., science, research, neurology, physiological, atom). Furthermore, responses that speculated on the ability of science to someday overcome mortality were categorised as *Potential of science*. Finally, we created the category *Ambiguous* for responses that were difficult to interpret or did not correspond to the criteria of any category. All the categories are listed in Table 1. The detailed coding instructions are archived in the Supplementary Material.

**Table 1.** The Categories of the Coding Template.

Category	Score	Meaning
Continuity social	0–2	The respondent expresses that humans or some part of them exists after death socially (in other humans or communities).
Continuity close others		The respondent expresses that humans or some part of them exists after death in people that personally knew the deceased, e.g., in memories of friends or family.
Continuity offspring		The respondent expresses that humans or some part of them exists after death in their offspring.
Continuity societal		The respondent expresses that humans or some part of them exists after death in their societal contributions, e.g. through their work.
Continuity mind/consciousness		The respondent expresses that the human mind/consciousness exists after death.
Continuity soul		The respondent expresses that the human soul or part of it exists after death.
Continuity other religious/New Age		The respondent expresses that humans or some part of them exists after death and applies terminology that is often associated to religion or New Age beliefs (excluding soul).
Continuity same/new body		The respondent expresses that humans or some part of them exists after death in a [human or animal] body.
Continuity natural laws		The respondent expresses that humans or some part of them exists after death and expresses this as a law of nature or justifies this view with a natural law, such as the regularities in physics or biology.
Continuity other		The respondent expresses that humans or some part of them exists after death but does not specify his/her view, or the view does not fit the criteria of any other continuity category.
Annihilation body/bodily functions	0–2	The respondent expresses that the human body or bodily functions cease/annihilate after death.
Annihilation mind/consciousness		The respondent expresses that the mind/thought/consciousness annihilates after death.
Annihilation no distinction		The respondent states that humans or some part of them is annihilated after death but they do not distinguish between the mind and the body.
Annihilation life metaphor (e.g. sleep, before birth)		The respondent describes annihilation after death with a life metaphor, such as deep sleep.
Science	0–1	The respondent mentions a science-related term (e.g., “science”, “research”, “biology”, “physiological” or “pharmacological”).
Potential of science		According to the respondent, science and/or technology might offer continuity after death as science/technology develops.
Death good/beneficial		The respondent expresses that there is something good, e.g., comforting or beneficial, about death or dying.
Conflicted		The respondent expresses that they experience conflicting feelings or thoughts on what happens after death.
Continuity comfort		The respondent expresses that a continuity belief is comforting (for themselves).
Continuity rejection		The respondent mentions continuity after death only to reject it. The responses were not coded in the continuity categories.
Ambiguous		It is difficult to read what the respondent means or the response does not fit the criteria of any other category.

*Note*. Annihilation and continuity categories were rated on the scale 0–2 (0 = does not fit the criteria, 1 = ambiguous wording such as “might”, 2 = fits the criteria) and other categories on the scale 0–1 (0 = does not fit the criteria and 1 = fits the criteria).

During the analysis, we noticed that some participants stated their response in a more conditional manner, while others seemed assured in their views. Additionally, a respondent might express one belief with certainty but hold another less firmly. To take this into account, we coded the annihilation and continuity categories on the scale 0–2, when 0 = no belief, 1 = hesitant wording and 2 = belief. For instance, one of the respondents stated that after death “[w]e cease to exist in the human form. Insofar as everything is ‘conscious’ to a certain extent, consciousness may continue in an altered state of being that is beyond the human imagination” (P19). This response was assigned the following codes and values: Annihilation no distinction 2, Continuity mind/consciousness 1.^11^

## Results

In the following sections, we describe the results. First, we introduce the frequencies of responses that refer to annihilation and/or continuity after death, followed by frequencies of the categories that mapped attitudes and the role of science in afterdeath beliefs. Secondly, we present our analysis of the response patterns. We also examined the relationship between the response categories and demographic information (gender, age, education, work affiliation to research, religious affiliation, self-described spirituality and God belief). The respondents’ age, education, and current or prior work in a research institution were not related to afterdeath beliefs. However, there were some differences in responses across other demographic variables and we describe these in the final section on the results.

### Frequencies of Annihilation and Continuity Beliefs

The content of the responses was explored by investigating which beliefs were mentioned most frequently. Overall, the majority of respondents emphasised that something annihilates upon death; for example, 76% (293 participants) mentioned some kind of annihilation when asked what happens to humans after death (response categorised as Annihilation no distinction, Annihilation body, and/or Annihilation mind). On the other hand, 35% (135) of respondents endorsed some kind of continuity in their response on what happens after death. A similar pattern could be found in the responses concerning human (in)finity, as 82% (316) mentioned annihilation and 37% (143) endorsed secular or supernatural continuity after death. It should be noted that the responses most likely do not contain *all* the afterdeath beliefs of the participants. For instance, even if some respondents did not mention annihilation, they might still agree that something of human existence ends in death; however, these participants have focused on something else in their response. Overall, 89% (345) mentioned some kind of annihilation in at least either of their responses and 53% (205) described continuity of humans (or some part of them) after death.

The most frequently mentioned view on what happens after death was annihilation without distinction between the mind and the body (60%, see Table 2). Many simply described that humans cease to exist or that nothing happens after death. However, some went on to describe continuity after they had mentioned annihilation without a mind–body distinction.

**Table 2.** Most Frequently Mentioned Categories on Annihilation and Continuity (Percentage and Number of Respondents, N = 387).

Order	Category	Endorsement % (*n*)	Uncertain endorsement % (*n*)
1	Annihilation no distinction	60% (234)	1% (5)
2	Annihilation body/bodily functions	55% (214)	0% (1)
3	Annihilation mind/consciousness	38% (146)	1% (5)
4	Continuity social	24% (92)	4% (15)
5	Continuity natural laws	20% (79)	3% (10)
6	Continuity close others	19% (73)	4% (15)
7	Continuity societal	17% (65)	1% (3)
8	Continuity religious/New Age	14% (53)	2% (7)
9	Continuity soul/spirit	8% (31)	4% (15)
10	Annihilation life metaphor (e.g., sleep)	6% (23)	0% (1)

*Note.* Respondents who mentioned the category with the same score in both questions were only calculated once.^29^

The second most frequently mentioned view was annihilation of bodily functions, as this was described in one or more responses by 55% of participants. Many stated that after death one’s body decomposes. Annihilation of the body was often paired with the third most frequently mentioned category, annihilation of the mind, consciousness or thought (38%). As one respondent stated “[w]hen we die the brain functions cease; therefore, all thinking stops” (P765). However, not all participants were eager to link bodily decay to annihilation of the mind. Some mentioned bodily demise but found it more difficult to decipher what happens to the consciousness. One respondent stated, “[a]t least our physical body decomposes and disintegrates. Disappears. Consciousness is a tough one. I hope that it continues living in some way […]” (P1284). Overall, the respondents were more assured in their annihilation beliefs and expressed uncertainty mainly concerning continuity after death (see Table 2).

The fourth most frequent afterdeath belief was continuity in social relations and legacy, which was mentioned by 24% of participants (28% when we include uncertain responses). The participants often described that although something ends in death, people or parts of them continue in memories. Some further specified that humans exist in the memories of loved ones, family or friends (‘Social continuity close others’). Others described continuity in societal legacy, such as scientific work, art or the impact a person had on the environment; these responses were also categorised as ‘Social continuity societal’. Some mentioned both kinds of social continuity, as the respondent who described what happens after death in the following manner:

The body starts to decompose. [My] own life continues in children and close relatives. Humans remain in memories of others only as long as they [the others] live. If one has accomplished something more permanent, like a book or a house, people can remember you longer. I think of myself as a link in a never-ending chain that reformulates itself, with the eldest parts slowly disappearing from sight. (P2160)

Many wrote that merely *a part* of the person remains in memories; therefore, humans continue only in a limited sense. The respondents that described social continuity along these lines often defined existence through consciousness, making continuation in memories a pale substitute for biological life. As one respondent stated, “[w]hen a person dies, they no longer exist. The history of the human can live on in the memories of loved ones for some time but the story of the deceased will not continue” (P2664). Another respondent suggested that upon death, the person does not disappear, as

[…] their essence, looks, experience of their laughter, temperament and smell has transferred to the memory of many living humans and continue their life in the memory of other people for a few more generations. In fact, nowadays perhaps even forever to the extent that these features have been uploaded to Cloud storage around the world […] (P2678).

However, the same participant also emphasised annihilation in death, stating that even if someone lived on in memories “[…] the person’s unique consciousness, tied to the unique body, can never reoccur as a subjective experience […]”. On the other hand, some placed higher value on continuing in social bonds. As one respondent wrote, “[…] humans can live in memories as long as someone remembers them. In this sense, the community is more important than the individual” (P2011).^12^

Another frequently mentioned continuity belief was continuation in natural laws, described by 20% (79) of the participants. Also in these responses, continuity after death retained merely *parts of* humans. Many referred to a process the respondents call the circulation of nature or life. Some discussed death as a return, or more specifically that “the ingredients that we consist of return back to the circulation of life” (P2036). In most of these views, the individual is annihilated and the body remains after death. Through the decomposing process, annihilation enables continuation of more long-lasting parts in nature.^13^ Unlike in the descriptions on social continuity, the physical remnants do not retain the individual persona. As one participant wrote, “[a]fter death the consciousness and the persona cease to exist and the body ends up like all organic material. We therefore continue forward in the circulation of nature” (P348). Some respondents mentioned atoms in an analogous manner stating that although the consciousness annihilates, the atoms continue. Several respondents also discussed the continuity of energy, referring to thermodynamics and the law of conservation of energy. Some specified that energy “leaves” the human body upon death. As the law of conservation of energy posits that energy does not disappear, the respondents associated this with the continuity of human parts or even the human spirit. For instance, one participant wrote,

I think that everything here is energy, originating from the Big Bang. Physically we consist of stardust, as any other matter here on Earth. The body decomposes or burns, the mind and the spirit are released. Energy changes its form but surely [or: as we know] its quantity is constant. The energy that remained in a human is released; [it] changes and proceeds. (P1844)

Several respondents suggested that if the body decomposes or otherwise “returns” to nature, its parts will form new life. Some discussed the forms that human parts may eventually take, as one participant described human (in)finity:

[…] what humans are made of circulates on in some form. When pieces, atoms etc., reunite a human is reverted but not as the same but as different. The building blocks [*rakennusaineet*] can also form something other than a human … for instance, an animal or a tree. (P2108)

Like social continuity beliefs, the importance that the respondents placed on continuity in natural laws varied. Many paired individual existence with consciousness and separated this from the nonindividual continuity in nature. In the words of one participant, even if atoms are infinite “an individual human is not only atoms” (P1076).

Although most respondents in our science-oriented sample referred to annihilation and/or nonsupernatural continuity after death, some endorsed an afterlife that could be viewed as religious or spiritual in a traditional sense (‘Continuity religious/New Age’). However, these beliefs were less frequent and only mentioned by 14% (53) of the respondents. In the responses, participants often referred to “God” or “Heaven”. Some discussed the body and the spirit separately, as in the following description on what happens after death:

Physiologically we probably just decay and, if buried, decompose. Spiritually—and this is just my own view—we continue [our] life somewhere else. I do not consider myself as religious but the religious teaching I got in my childhood and youth have maintained an idea of Heaven. […] (P1004)

Although the author of the quote did not regard themselves as religious, some explicitly identified with religion. For instance, another participant discussed afterdeath by describing bodily decay and concluding that “[…] I am religious and believe in an afterlife of the soul in Heaven. However, this does not exclude that I believe in science” (P23). Several respondents mentioned the soul and (other) religious or spiritual belief in the same response. Overall, immortality of the soul or spirit was expressed by 8% (31) of the respondents. Additionally, as with other continuity categories, some leaned towards an afterlife of the soul but expressed some uncertainty (4%, 15). Other kinds of supernatural continuity were even less endorsed; only 4% (15) responded that humans continue after death in an unspecified place or form and 3% (12) expressed belief in the continuity of mind or “consciousness” after death.

### Frequencies of Other Categories

In addition to descriptions on what remains after death, respondents also specified their relation to continuity beliefs in other ways (see Table 3). Most frequently, the respondents emphasised that they do not believe in continuity (22%, 86). This manifested mainly as rejection of supernatural beliefs and (other) views that are often perceived as religious. Many underlined that they do not believe in “Heaven etc.” (P1694) or an afterlife. As one respondent wrote, “I don’t believe in life after death. When a person dies, bodily functions cease and consciousness disappears. The person ceases to exist in all possible ways” (P174). However, rejecting a supernatural afterlife did not necessarily exclude endorsement of continuation of other kinds. For instance, one participant wrote that they do not believe in any other reality, yet the deceased individuals “[…] are present in the thoughts of those who still live, and I think that in a way it is very valuable life after death” (P806). Therefore, the responses coded as rejection of continuity mainly criticise *supernatural* or religious belief, rather than secular forms of continuation.

**Table 3.** Frequencies of Attitude Categories and the Categories Mapping the Role of Science in Responses (Percentage and Number of Respondents, N = 387).

Category	Endorsement % (*n*)
Continuity rejection	22% (86)
Conflicted	9% (34)
Death good/beneficial	8% (31)
Continuity comfort	3% (13)
	
Science-related terminology	35% (135)
Potential of science	5% (19)

On the other hand, 9% (34) of the respondents expressed conflicted views, in which they often wanted to believe in supernatural (or similar) continuity after death. For instance, one respondent wrote that they would “[…] very much want to believe in going to the ‘home in Heaven’ but unfortunately reason stumps [this] belief” (P1763). Also some others divided their experiences into conflicting parts (e.g., reason and wishes) that could come to different conclusions. As one participant stated, after death “[t]he electronic brain functions cease, consciousness flames out. Against cold logic, I would like to believe in the law of thermodynamics, which states that energy never disappears from the system but merely changes its form” (P1839). However, conflicted responses also reflected uncertainty; some respondents felt torn, as they did not know what to believe about human’s existence after death. One participant described this feeling as being “[…] undecided, between two options. Either consciousness simply flames out or we get to return to our loved ones” (P2533).^14^ However, whereas some felt conflicted or overwhelmed, others found a “silver-lining” in death and described mortality as comforting or beneficial (‘Death good/beneficial’8%, 31). This category contained several somewhat differing beliefs. Death as comforting was mostly related with the expectation of a pleasant state that follows. For instance, one respondent expected rest and perhaps a reunion with close others:

I believe, and hope, that after death we get to rest. I cannot guess the exact nature of that rest, but in the best case scenario it is like coming home after a long, hard day and falling asleep in the warm gloaming with your loved ones. (P1105)

Similarly, some emphasised that death marks the end of suffering, whether it be a pleasant feeling or lack of experience whatsoever. Another line of responses saw positive aspects in death mainly in how bodily decay benefits nature, e.g., by producing nutrients for new life. Similarly, some described their preference for disposal of the body as merging into nature was preferred to artificial preservation. For instance, one respondent wrote about death in the following manner:

[…] After that [death] the human body remains; it disintegrates into chemical elements, either slowly or with accelerated speed (cremation). At its best, the ashes will not be packed in a non-biodegradable urn but they are spread back to nature, where they can still benefit the ecosystem. […] (P489)

More than one third of the respondents applied science-related terminology in their depictions of death and what follows. However, few respondents discussed the ability of science to eventually eliminate human mortality, e.g., with technological enhancement of the body and/or mind uploading (5%, 19). Overall, the meaning of science-related terms in afterdeath can better be understood by examining what kind of responses applied them. Next, we will describe overall patterns on how afterdeath beliefs, attitude categories and science-related terminology were associated in the responses.

### Response Patterns

To explore which beliefs were often mentioned together, we conducted a principal component analysis on the responses. For the analysis, we formulated variables for each category that contained the sum value of the participant’s responses for both questions analysed in this study. However, we excluded the category ‘Continuity close others’ from the analysis, as it was strongly correlated with the overall category ‘Social continuity’. The analysis isolated eight components with eigenvalues >1.0 that accounted for 64% of the variance.^15^ All the components are listed in Table 4. Here, we will briefly describe the five patterns in the responses that explained the most variance in the data.

**Table 4.** Component Matrix of the PCA on Response Patterns.

	C1	C2	C3	C4	C5	C6	C7	C8
Eigenvalue	2.39	1.89	1.68	1.50	1.39	1.12	1.09	1.03
% of the variance explained	13%	10%	9%	8%	7%	6%	6%	5%
Annihilation mind	**.591**	.207	**.415**	−.366	.017	−.139	.123	−.008
Continuity religious/New Age	**−.543**	**.497**	−.027	.048	−.223	.195	.072	−.097
Annihilation body	**.529**	**.475**	.149	−.361	.085	−.055	−.013	.002
Annihilation no distinction	−.198	**−.782**	−.157	.194	.025	.236	.081	.043
Continuity soul	**−.414**	**.510**	.043	.201	−.123	.229	.201	−.039
Continuity mind	−.230	.344	−.052	.113	−.040	−.072	−.224	.341
Continuity same/new body	−.292	.328	−.012	−.118	−.096	.195	.324	.002
Continuity social	**.601**	.162	**−.668**	.150	−.103	.022	.052	.007
Continuity societal	**.572**	.121	**−.643**	.130	−.210	.117	.090	.030
Continuity natural laws	.169	.054	**.438**	**.651**	.100	−.142	.017	−.116
Continuity offspring	.155	.104	−.142	**.501**	.290	−.162	.021	−.059
Science	.388	.140	**.458**	**.474**	.017	.284	−.216	.114
Conflicted	−.078	.074	−.169	−.021	**.782**	.086	−.055	.138
Continuity comfort	−.127	.185	−.122	−.150	**.667**	.157	.090	.263
Potential of science	.271	−.051	.156	−.037	−.196	**.605**	−.340	**.418**
Death good/beneficial	.127	.028	.128	.315	−.059	−.239	**.573**	.397
Continuity other	−.232	.129	−.118	.022	−.121	**−.524**	**−.536**	.205
Annihilation life metaphor	−.013	−.305	.149	−.202	−.169	−.159	.257	**.554**
Continuity rejection	.245	−.217	.241	−.154	.122	.113	.068	−.324

*Note*. Component loading >.400 in bold. The PCA was based on the correlation matrix.

The first component reflected a cluster of responses that suggested that humans may live on in social relations and legacy (‘Continuity social’, ‘Continuity societal’), although the consciousness ceases to exist (‘Annihilation mind’) and the body decays (‘Annihilation body’). In this component, participants were less likely to mention belief in the soul or other religious afterlife (‘Continuity religious’, ‘Continuity soul’). Instead, an emphasis on religious belief comprised the second component. In this pattern, the religious responses often entailed that death signifies the annihilation of the *body* (‘Continuity religious’, Continuity soul’, ‘Annihilation body’). As one respondent stated, “[o]ur body decays and decomposes. We no longer actively influence the world. I believe and hope that after death there is the hope of resurrection […]” (P526).^16^ Similarly, the component was negatively associated with descriptions of annihilation that did not separate between the mind and the body (‘Annihilation no distinction’), e.g., that “[…] there is nothing” after death (P312). The third response pattern reflected responses where after death, parts of humans continue in nature (‘Continuity natural laws’), although the mind ceases to exist (‘Annihilation mind’). The participants who described death in this manner often applied science-related terminology (‘Science’). As one respondent wrote,” [b]iologically we start to decompose and continue in the circulation of nature. The consciousness flames out instantly […]” (P2766).^17^ In this cluster of responses, those who mentioned symbolic continuation in nature were less likely to describe continuity in memories or legacy (‘Continuity social’, Continuity societal’).

Also the fourth pattern comprised descriptions of something of humans remaining in physical or biological processes (‘Continuity natural laws’). More specifically, the component reflected descriptions of continuation in descendants (‘Continuity offspring’), as some stated that *genes* might extend the human existence (‘Science’). As one respondent put it, “I think the existence of a human can be infinite. Our genes are passed forward to our children” (P1307). Finally, the fifth component comprised the respondents’ accounts that belief in (mainly religious) continuity would be a comforting way to tackle the mortality of self or others (‘Continuity comfort’); yet they were not able to rely on it but reported ambivalent feelings or thoughts (‘Conflicted’), as discussed in the descriptions of frequencies of the categories.^18^

Although the components do not describe patterns in the data exhaustively, they shed light on which categories could be grouped together in a model that retains the most significant differences between the respondents. To summarise, all the components contained a category that either exceeds the finality of death (continuity categories), discusses whether death could be deferred in the future (‘Potential of science’), contains the wish to believe in an afterlife (‘Continuity Comfort’) or engages in benefit-finding (‘Death good/beneficial’) (see Table 4). Therefore, the respondents mainly seemed to differ in their strategies to exceed or make meaning of mortality. The top three response patterns also loaded onto annihilation categories (> .400). Among these, the component that comprised religious belief clustered with the annihilation of mere body, whereas patterns that reflected nonsupernatural continuity comprised the cessation of the mind.

### The Role of Unbelief and Gender

As mentioned, there were some differences across demographic groups in afterdeath beliefs. Firstly, women were more likely to express conflicted feelings over what happens after death than other gender groups (*p* = .054, Fisher’s Exact Test, FET) and less likely to mention that the mind ceases to exist (*p* = .002, FET). Furthermore, gender was associated with describing the annihilation of the body; a relationship we cannot quite decipher (Kruskal-Wallis *H*[2] = 7.585, *p* = .023).^19^

However, the demographic variables the most associated with afterdeath beliefs were religious affiliation, self-described spirituality and belief in God. God belief was associated with descriptions of conflicted feelings about afterdeath (*p* = .035, Fisher’s Exact Test), as those unsure of their theism/atheism mentioned inner conflict more frequently than others. Theists and those unsure of their God belief were more likely to endorse a supernatural afterlife, such as the immortality of the soul, than atheists. Furthermore, secular (nonsupernatural) continuity beliefs were most often mentioned by those who did not believe in God. More specifically, continuity in natural laws appeared as mainly an atheist belief in our sample (Kruskal-Wallis *H*[2] = 6.915, *p* = .032) and also the endorsement of social continuity was negatively associated with God belief (*p* = .033, FET). Moreover, God belief was associated with the annihilation categories, as atheists were most likely to refer to mortality of the consciousness (*p* < .001, FET), bodily annihilation (*H*[2] = 14.224, *p* = .001), and annihilation without distinction between the mind and the body (*H*[2] = 23.238, *p* < .001).^20^ On the other hand, theists applied less science-related terminology in their views on afterdeath than atheists and those unsure of their God belief (*p* = .037, FET). The relationship between the respondents’ religious affiliation and the belief categories followed a somewhat similar pattern, although religiosity was less associated with conceptions on after death than God belief. Similar to God belief and religiosity, self-described spirituality was positively associated with supernatural belief categories.

## Discussion

In this study, we explored how science-oriented individuals describe what happens to humans after death and whether identification with science entails views on “secular death”, in which an individual is annihilated as the bodily functions cease. In our sample, science-oriented Finns mainly believed in this kind of a secular conception of death. However, most also expressed that *something* of humans remains post-mortem. The responses on continuity after death contained supernatural and naturalistic (nonsupernatural) views, and these remained as somewhat distinct sense-making strategies. Supernatural afterlife was more likely mentioned by God-believers, and it mostly applied religious terminology (e.g., referring to the immortality of the soul instead of the mind). On the other hand, nonsupernatural continuity was more likely endorsed by respondents who were atheists or unsure of their God belief.^21^ Furthermore, unbelievers were more likely than theists to mention science-related terminology. Science-related terms were applied to describe annihilation–but also in discussions on continuation, e.g., in nature through bodily decay (see also Manning, 2018). These views are interesting, as studies have often associated the biological conception of death with *lack* of continuity (or more precisely, cessation of bodily and mental states; see Georgiadou and Pnevmatikos, 2019; Harris & Giménez, 2005). However, as we extend the investigation of biological views beyond the level of individual functionality and explore people’s reflected notions, biological knowledge (or interpretations of it) can facilitate the belief that upon death, humans merge into something meaningful that outlasts them (see also Manning, 2018; Toscani et al., 2003).^22^ In addition to the nonindividual continuation in nature, some respondents discussed the possibility of continuity in memories of others–a symbolic form of existence which, to a limited extent, could retain parts of individuality.

Previously, naturalistic continuation has mainly been discussed in the literature on symbolic immortality, and our results align with prior suggestions that humans may gain a sense of continuation, e.g., from nature or social legacy (e.g., Florian & Mikulincer, 1998; Lifton, 1973; Vail et al., 2010). However, prior empirical investigations on whether people actually endorse or discuss symbolic immortality (and who does so) have been scarce. For instance, few studies have described people’s secular notions of living on in memories (however, see Coleman & Arrowood, 2015; cf. Jonsson & Aronsson, 2015).^23^ Also the role of belief in natural laws in (atheists’) views on afterdeath has received very little attention (although see Manning, 2018; Toscani et al., 2003).^24^ One explanation might be the focus of prior studies on beliefs that retain the consciousness, leaving a gap in the research for more secular afterdeath imagery. For instance, Burris and Bailey (2009, p. 174) argue that “if consciousness does not survive the death event, then the perseveration of identity and physicality is not meaningful: the self experiences Annihilation”. However, even if some scholars deem nonsupernatural continuity insignificant, our results suggest that naturalistic annihilation/continuity beliefs might still be meaningful for some science-oriented individuals.^25^

Overall, as some respondents endorsed religious and/or spiritual afterlife beliefs in their descriptions (e.g., belief in Heaven), our findings add to the body of research suggesting that scientific and religious orientations are not mutually exclusive, as people apply both scientific and religious explanations to make sense of death (Legare et al., 2012; Legare & Shtulman, 2018). However, the results also suggest that secular sense-making is of primary importance in questions concerning death for science-oriented individuals in the Finnish context (cf. Walker, 2000).

Furthermore, the findings on unbelievers’ nonsupernatural views on continuation after death raise the question whether these continuity accounts may be secular equivalents for afterlife belief, in the sense that they might serve similar needs (Florian & Mikulincer, 1998; Vail et al., 2010; cf. Farias, 2013) and/or reflect the same cognitive tendencies (cf. Hodge, 2018) as religious belief. Also this question is bound with the literature on symbolic immortality–and one that our study is not apt to answer. Next, we will review this and other limitations of our work and suggest directions for future research.

### Limitations and Future Questions

The limitations of our study could be discussed in three parts. Firstly, although we assumed, partly based on the belief replacement hypothesis, that the respondents might also express forms of nonsupernatural continuity, our study did not decipher whether naturalistic continuation might serve the same psychological functions as supernatural afterlife belief or whether continuity in general holds a similar importance for atheists as it does for God believers. Recent findings suggest that the hope of secular immortality can function similarly to afterlife belief for less religious individuals (Lifshin et al., 2018; Vail et al., 2020). However, studies also indicate that some atheists derive comfort and/or an enhanced sense of meaning from the finity of life, at least according to their self-reports (Coleman et al., 2016; Haimila, 2020). We hope that future research sheds light on this discussion. Studies could further investigate the significance of the forms of symbolic immortality described here (and as discussed by scholars such as Lifton (1973) and Florian and Mikulincer (1998), contra, e.g., Tracy et al., 2011), for instance, whether endorsement of living on in memories or merging into nature alleviates the effects of death reminders (Florian & Mikulincer, 1998; Wojtkowiak and Rutjens, 2011). If naturalistic continuity is more than “a footnote” for atheists, it might be beneficial for coping during bereavement, as has been suggested for afterlife belief (Smith et al., 1992).^26^

Secondly, although we aimed for a data-driven analysis, we began with a researcher-based divide between annihilation and continuity. Overall, we found this division as functional. However, it did provide some challenges for coding. More specifically, continuity in natural laws was at times difficult to categorise, as the responses on circulation of nature often focused on annihilation and *some* also referred to continuity, often crossing our annihilation—continuity divide. Therefore, although our coding focused on continuity in circulation of nature, based on the data the overall concept might mainly *overlap* with continuation; the aspect of annihilation in circulation might be equally important (for similar notes on belief in “cycle of nature”, see Butters, 2021, p. 175).^27^

Finally, it should be noted that although we discuss “the beliefs” of the participants, the results mainly reflect what the respondents thought about while answering our questions and experienced as worth mentioning. It is therefore difficult to say to what extent similar descriptions extend to other contexts. Would the participants describe annihilation and/or continuity in similar ways in other situations? It is possible that as the question “What happens to us after death?” is often associated with religious afterlife belief, the question in itself, on the one hand, might increase the mentions of continuity of some kind and, on the other hand, produce reactions in which participants disassociate themselves from traditional afterlife belief (responses that we categorised as rejection of continuity). However, as we are not equipped to examine this in the current study, we hope that future research will extend to articulated and/or enacted death conceptions in more varying contexts (cf. Taves et al., 2018).^28^

## Conclusion

According to Gire (2014, p. 2), “death imitates life”, in the sense that cultural surroundings impact our views on dying. This study explored the afterdeath beliefs of science-oriented Finns. In the analysis, we investigated the responses of 387 Finnish adults recruited via pro-research organisations. According to the results, science-oriented Finns mainly believed that consciousness annihilates as bodily functions cease. However, many also expressed that humans continue in some form after death. The continuity beliefs were mainly naturalistic (nonsupernatural) as they mostly described continuation that merely retains parts of the deceased, e.g., in memories or circulation of nature. The results suggest that also secular and science-oriented afterdeath conceptions may contain a sense of continuity that some people endorse in (or alongside) their annihilation beliefs. Naturalistic continuity after death was mainly mentioned by atheists, while theist respondents relied more on supernatural afterlife. However, further research is needed to examine the importance of annihilation and continuity beliefs among different demographic groups and to investigate whether secular continuity after death can serve similar psychological functions as afterlife belief. The current research provides knowledge on the versatility of contemporary views on afterdeath in the Finnish context.

## Supplemental Material

sj-pdf-1-ome-10.1177_00302228211038820 - Supplemental material for A Sense of Continuity in Mortality? Exploring Science-Oriented Finns’ Views on Afterdeath Click here for additional data file. Supplemental material, sj-pdf-1-ome-10.1177_00302228211038820 for A Sense of Continuity in Mortality? Exploring Science-Oriented Finns’ Views on Afterdeath by Roosa Haimila and Elisa Muraja in OMEGA-Journal of Death and Dying

## Notes

1 Furthermore, people apply also other natural and supernatural explanations, that may (or may not) align with scientific or religious content, such as folk biological accounts (see Hodge, 2018; Legare & Shtulman, 2018).

2 Studies in the cognitive science of religion on reasoning about death have found that some endorse continuity of mental states also in secular settings–albeit people tend to perceive supernatural continuity more after religious primes (Astuti & Harris, 2008; Harris & Giménez, 2005; cf. Watson-Jones et al., 2017). This literature has shed less light on *reflected* afterdeath beliefs and focused on more implicit views, e.g., the endorsement of supernatural continuity without an explicit belief in an afterlife (Bering, 2002). Despite the frequent use of student samples in psychology of religion, to our knowledge few have investigated reflected afterdeath beliefs in secular contexts, such as pro-science organizations. As an exception, Walker (2000, p. 5) measured the “secular eschatologies” of American healthcare workers and found that most believed in an afterlife.

3 It should be noted that in prior literature, the term “symbolic immortality” has been applied in various ways. For instance, in terror management theory (TMT), symbolic immortality can refer to 1) the ability of worldviews and beliefs that do not entail a promise of an afterlife to buffer the effects of death reminders, a form of symbolic immortality that does not necessarily entail a logical connection to exceeding the finality of death (e.g., increased trust in evolution following mortality salience, see Tracy et al., 2011), and 2) ways to exceed the finitude of life that contain an explicit connection to continuity after death, e.g., living on in one’s offspring (e.g., Vail et al., 2010; cf. also Florian & Mikulincer, 1998; contra Dechesne et al., 2003). The latter way to discuss symbolic immortality has also been proposed by Lifton (1973).

4 Toscani et al. (2003) interviewed eight Italians, including four unbelievers in a traditional afterlife.

5 However, it should be noted that in Finnish, the word “science” is often wider in scope than in the Anglo-American context and, for some, includes also the humanities and/or the social sciences (see Haimila, 2020). Therefore, the Finnish word for “science” resembles equivalent terms in other Nordic languages (Hansson, 2015, p. 15).

6 In the recruitment ad, we mentioned “worldviews that appreciate science” or “hold science in high regard”.

7 Both questions were applied to gain further details on participants’ views, as it was expected that some might answer the first question in a brief manner.

8 Moreover, some described the experience of annihilation by comparing it to the state before birth (see also Coleman et al., 2016). Although participants that discuss prenatal existence do not refer to life but rather some state preceding it, we also categorized these responses to the category of life metaphors.

9 More specifically, we first established the categories ‘Continuity close others’ and ‘Continuity societal’ to capture the responses that described continuation in the memories of others. However, some occurrences were difficult to code since many did not specify whether the deceased live on in the memories of loved ones or people more generally, as in the case of celebrities. Therefore, all continuity in social impact was coded to the main category ‘Social continuity’ and further specified with close/distant proximity, when applicable.

10 Furthermore, some studies suggest that people conceptualize the mind and the soul differently (Harris & Richert, 2008; in the Finnish context, see Lindeman et al., 2015).

11 Another participant wrote that: “[…] Once the brain functions cease the material substrate of consciousness will perish. If the consciousness is based solely on brain functions, it seems highly unlikely that any information would be transferred anywhere else or otherwise remain. However, if the brain in some way interacts with some conscious or “spiritual” dimension, it is possible that some information proceeds outside the human body or otherwise remains. This information could be called e.g., the Soul […]” (P680). This response was categorised with the following codes and values: Annihilation body/bodily functions 2; Annihilation mind/consciousness 1; Continuity soul/spirit 1 and Continuity religious/New Age 1.

12 Another described continued human existence through legacy or societal impact: “[…] If you are an artist, author or some historical figure of importance or for instance our ancestor Lucy that was found in Africa or iceman Ötzi, existence can continue for hundreds of thousands of years. Then existence is not the existence of individual’s own consciousness but shared cultural experience of a person that once was” (P2594).

13 As one respondent stated, “[a]fter death we decompose so our ingredients continue their circulation in the universe” (P796).

14 Also other respondents discussed the importance of social relations in these responses. As one participant wrote, humans “[…] probably cease to exist. But for the sake of my own psyche, I must think that my late mother is somewhere safe (in a safe place)” (P2097).

15 Further rotation of the model with Varimax (Kaiser normalization) yielded more simplified components. In this footnote, we list the content of these components, in case the reader would like more information on the rotated model: C1: Annihilation mind, Annihilation body, Annihilation no distinction [R]; C2: Continuity social, Continuity societal; C3: Continuity religious/New Age, Continuity soul, Continuity same/new body; C4: Continuity natural laws, Science, Continuity offspring; C5: Continuity comfort, Conflicted; C6: Continuity other, Continuity mind; C7: Potential of science, Science; C8: Death good/beneficial, Annihilation life metaphor. Somewhat similar patterns were found also with Oblimin rotation method. As the two questions the participants answered were formulated differently, we also analysed the responses to both questions separately to check for differing patterns between categories.

16 On resurrection, the respondent continued that “[i]ts essence is a mystery, but is a comforting thought that my restless heart would gain peace in the Christ […]” (P526). As a further note, the response was not coded as ‘Continuity same/new body‘as we were not entirely sure what exactly the respondent referred to as “resurrection”.

17 Some other science-related terminology in similar responses included “infinite components, such as the subatomic particles” (P2349) or “chemical elements”, that are “eternally circulating” (P2090).

18 The remaining three components accounted for the least differences between respondents and comprised of afterdeath beliefs that were mentioned less. The sixth component reflected some of the responses that discussed the potential of science at some point to eliminate mortality concerns–these were less likely to contain supernatural continuity without religious terminology. In the seventh component, the aforementioned (lack of) afterlife belief was associated with accounts on death as good or beneficial. In the eighth component, speculation on the potential of science to conquer human mortality was coined with comparing death with something that happens during life (e.g., deep sleep) or precedes it (the state before birth), a possible strategy to make sense of nonexistence of the consciousness.

19 More specifically, there was a significant difference between men and respondents who chose “Other/I don’t want to say” (*p* = .022), with “Other/I don’t want to say” group mentioning bodily annihilation the most (mean rank 258.43) and men the least (mean rank 184.72). The association between annihilation of the body and gender might be related to the small group size in the “Other/I don’t want to say” category (*n* = 15). The “Other/I don’t want to say” group was less spiritual than other gender groups (*H*[2] = 6.355, *p* = .042, mean rank women = 205.15, men = 185.23, and Other/I don’t want to say = 143.27). However, there was only a slight negative correlation between spirituality and descriptions of bodily annihilation (*r_s_* = –.15, *p* = .004).

20 Lack of God belief was also related to the rejection of (mainly religious) continuity (p < .001, Fisher’s Exact Test).

21 Moreover, religious belief and most frequently mentioned forms of nonsupernatural continuity seemed to entail different kinds of annihilation views; endorsement of religious afterlife clustered together with annihilation of (mere) body, whereas, in our PCA model, symbolic continuity was more likely paired with descriptions of the mind ceasing to exist.

22 In other words, whereas folk biological reasoning about death focuses on annihilation, reflected views on biological processes may, for some science-oriented individuals, complement this with one form of symbolic immortality (cf. Lifton, 1973).

23 Continuity in memories has also been discussed in the literature on continuing bonds or continued attachment (Klass et al., 1996; Jonsson & Aronsson, 2015), which some perceive mainly as a religious phenomenon (Benore & Park, 2004). Nevertheless, in our sample, living on in memories did not contain any continued experience of the deceased (see also Coleman & Arrowood, 2015; cf. Burris and Bailey, 2009).

24 However, some have noted the possible importance of somewhat similar beliefs for Finns more generally. In a recent representative survey, 26% of Finns at least somewhat agreed that after death, we “blend back into the universe” (Ketola et al., 2018, pp. 46–47). Furthermore, the return to the circulation or cycle of nature was also mentioned by some of the Finnish hospice patients interviewed by Butters (2021).

25 However, the respondents had a variety of ideas about what counts as *existence*. Many, like Burris and Bailey (2009), focused on the annihilation or continuity of conscious self and in these accounts other possible forms of continuity, if mentioned, were valued less (individualistic or egocentric perspective). Still, some determined existence also or primarily as socially defined (social perspective) and a few others focused on continued existence of smaller particles in a way that made annihilation of consciousness matter less (physical or biological perspective). However, our research design was not able to further investigate these varying views on existence; the importance of different types of annihilation and continuity beliefs could be explored in future studies (cf. Florian & Mikulincer, 1998).

26 The importance of the well-being and/or location of the decedent was evident in some of the conflicted responses that included the hope of an afterlife, and it is difficult to say whether secular continuity (which does not entail continuation of experience of the deceased) could alleviate grief in a similar manner. However, upholding social continuity might help maintain a sense of connection (cf. social death in e.g., Hodge, 2018; Koski, 2014) and expected continuation in nature might provide a way to perceive the deceased “somewhere” and as a part of something meaningful (cf. Jonsson & Aronsson, 2015, p. 53; Pyszczynski et al., 1999; Hodge, 2018).

27 During the template development, we considered categories for both ‘Continuity natural laws’ and ‘Annihilation natural laws’. However, this created challenges as many responses contained both and the boundary between these seemed slippery (and at times artificial) in the views on circulation of nature. However, other beliefs in ‘Continuity natural laws’ were more clearly focused on continuation. For instance, referring to thermodynamics usually entailed the hope of continuity of “energy” that some associated with a personal essence or soul.

28 Studies have found that people endorse continuation of mental states more in religious contexts compared to situations that remind them of science (Astuti & Harris, 2008; Harris & Giménez, 2005). Therefore, recruiting participants to a survey on “worldviews that appreciate science” might have decreased mentions of supernatural afterlife and bolstered secular death narratives.

29 The top ten categories listed in Table 2 were followed by ‘Continuity offspring’ (4% endorsement, 1% uncertain); ‘Continuity other’ (4% endorsement, 3% uncertain); ‘Continuity mind/consciousness’ (3% endorsement, 3% uncertain); and ‘Continuity same/new body’ (1% endorsement, 2% uncertain endorsement).
