# Supplementary material for: A Sense of Continuity in Mortality? Exploring Science-Oriented Finns’ Views on Afterdeath
Source: Omega (Westport). 2021 Aug 18;88(1):38–65. doi: 10.1177/00302228211038820 (PMC10568951; doi:10.1177/00302228211038820)
Supplement: sj-pdf-1-ome-10.1177_00302228211038820 - Supplemental material for A Sense of Continuity in Mortality? Exploring Science-Oriented Finns’ Views on Afterdeath [file sj-pdf-1-ome-10.1177_00302228211038820.pdf]

SUPPLEMENTARY MATERIAL

Contents

A. List of sources for participant recruitment 1

B. The control question 4

C. The coding instructions 5

D. The study questionnaire (in Finnish) 9

Bibliography 18

A. List of sources for participant recruitment

List of sources for participant recruitment (12/2019–2/2020). To ensure participants from research organizations would have the opportunity to participate, I first contacted 1) and 2), followed by 3).

1) Research organizations

**Name**	**Section**	**Field**	**Email and/or social media**
University of Helsinki	Faculty of Humanities staff: all sections	Humanities	Email
University of Helsinki	Doctoral School in Humanities and Social Sciences doctoral students	Humanities and Social Sciences	Email
University of Helsinki	Faculty of Medicine Research Programme Unit staff	Medicine	Email
University of Helsinki	Faculty of Science Chemistry staff	Natural sciences	Email
University of Helsinki	Faculty of Science Mathematics and Statistics staff	Natural sciences	Email
University of Helsinki	Faculty of Science Geoscience and Geography staff	Natural sciences	Email
University of Helsinki	Faculty of Science Physics staff	Natural sciences	Email
University of Helsinki	Faculty of Science Institute for Atmospheric and Earth System Research (INAR) staff	Natural sciences	Email
University of Helsinki	Faculty of Law staff	Social sciences	Email
University of Helsinki	Logopedics students	Medicine	Email
Aalto University	School of Science researchers, including doctoral students	Natural sciences	Email
University of Eastern Finland	Faculty of Health Sciences staff and doctoral students	Natural sciences	Yammer
University of Eastern Finland	Philosophical Faculty staff	Humanities	Yammer
Tampere University	Faculty of Information Technology and Communication Sciences	Natural sciences, Humanities, Social sciences	Email
University of Turku	Doctoral Programme in History, Culture and Arts Studies doctoral students	Humanities	Email
University of Turku	Study of Religion doctoral students and staff	Humanities	Email
University of Turku	Finland Futures Research Centre	Humanities, Social sciences	Employees' intranet
University of Jyväskylä	Faculty of Humanities and Social Sciences staff and doctoral students	Humanities, Social sciences	Email
University of Oulu	Faculty of Humanities doctoral students	Humanities	Email
University of Lapland	Faculty of Social Sciences staff	Social sciences	Email
VTT Technical Research Centre of Finland	Technical Research Centre staff	Natural sciences	Email

2) Associations affiliated to research organizations

Name	Additional information	Research organization	Field	Email list and/or social media
Mana ry	Student organization for social and cultural anthropology students	University of Helsinki	Humanities	Email
HAO ry	Student organization for subject teacher trainees	University of Helsinki	Humanities/Other	Email
Dilemma ry	Student organization for philosophy students	University of Helsinki	Humanities	Email
Matlu ry	Student organization for Faculty of Science students	University of Helsinki	Natural sciences	Email
Meridiaani ry	Student organization for astronomy students	University of Helsinki	Natural sciences	Email
Synop ry	Student organization for meteorology students	University of Helsinki	Natural sciences	Email
Vasara ry	Student organization for geology students	University of Helsinki	Natural sciences	Email
Status ry	Student organization for social psychology students	University of Helsinki	Social sciences	Email
Konstruktio ry	Student organization for students of social sciences	University of Helsinki	Social sciences	Email
Teema ry	Student organization for literature students	Tampere University	Humanities	Email
Lexica ry	Student organization for foreign languages students	Tampere University	Humanities	Email
Patina ry	Student organization for history students	Tampere University	Humanities	Email
Fiskus ry	Student organization for public financial management students	Tampere University	Social sciences	Email
Vostok ry	Student organization for journalism and mass communication students	Tampere University	Social sciences	Email
Interaktio ry	Student organization for students of social sciences	Tampere University	Social sciences	Email
Iltakoulu ry	Student organization for political science and international relations	Tampere University	Social sciences	Email
Kosmos Buran ry	Student organization for political science and sociology students	University of Lapland	Social sciences	Email
Fokus ry	Student organization for students of social sciences	University of Jyväskylä	Social sciences	Email
Vare ry	Student organization for students of archaeology	University of Turku	Humanities	Email
Asteriski ry	Student organization for computer science students	University of Turku	Natural sciences	Email
Hybridi ry	Student organization for science students	University of Turku	Natural sciences	Email
In Vitro Aboensis IVA ry	Student organization for biomedicine students	University of Turku	Natural sciences	Email
Black Swans - Futures Studies Students of Turku ry	Student organization for future studies students	University of Turku	Social sciences	Email
Data Guild ry	Student organization for data science students	Aalto University	Natural sciences	Email
Lämpövoimakerho, LVK Energy Technology student club	Student organization for energy technology students	Aalto University	Natural sciences	Email
Poligoni ry	Student organization for geoinformatics students	Aalto University	Natural sciences	Email

Social media recruitment 12–14/2/2020

3) Organizations that popularize and/or promote research [that were invited]

**Name**	**Additional information**	**URL**	**Medium**	**Following**
Yle Tiede	Finnish public media broadcaster's section for news on science and research. Yle Tiede retweeted the invitation 12/2/2020.	https://twitter.com/yletiede	Twitter	> 58 k followers
Tiede.fi	Finnish science magazine. The invite was posted 12/2/2020 on three discussion boards: 1) Biology and environment, 2) History, cultures, and society, and 3) Other science topics. The invitation raised active discussion in discussion board 3) Other science topics. Therefore, the invitation remained in the front page of Tiede discussion for the two-day period of social media recruitment.	https://www.tiede.fi/keskustelu	Internet discussion forum	
Skepsis ry	Finnish skeptics' association, a "scientific organization" that promotes a worldview "based on science and reason". Invitation was shared in the Facebook group 14/2/2020.	https://www.facebook.com/groups/skepsisry/	Facebook	> 23 k followers

4) Other organizations that shared the invitation in social media

**Name**	**Additional information**	**URL**	**Medium**	**Following**
Silakkaliike	A movement that aims to advance human dignity, nature, science, equality and non-discrimination and stand in opposition to fascism and climate change denialism (https://silakkaliike.fi/). Invitation was shared in the Facebook group 14/2/2020.	https://www.facebook.com/groups/Silakkaliike/	Facebook	> 28 k followers
Geek Women Unite! (Finland)	A networking group for those that identify as geek/geekish women. Invitation was shared in the Facebook group 14/2/2020.	https://www.facebook.com/groups/207664933742/	Facebook	> 5200 followers
Ursa ry	Finnish astronomy association. Invitation was retweeted 12/2/2020.	https://twitter.com/Ursa_ry	Twitter	> 3700 followers
Union of Freethinkers of Finland	Finnish freethinkers association that aims to advance the rights of non-religious in Finland and promote a science-based worldview. Invitation was shared 13/2/2020.	https://www.facebook.com/groups/vapariliitto/	Facebook	> 2400 followers
Areiopagi	Finnish journal on the intersections of natural sciences, philosophy and theology.	https://twitter.com/areiopagi	Twitter	> 800 followers
Young Academy Finland / Nuorten Tiedeakatemia	An association that promotes science and scholarships of young researchers.	https://twitter.com/yaf_fi	Twitter	> 400 followers
Jyväskylän Sirius ry	Astronomy association in Central Finland.	https://twitter.com/jklsirius	Twitter	> 100 followers
Geohouse / Geotalo/ Geohuset	"Home to geologists and archaeologists" at the University of Turku and Åbo Akademi.	https://twitter.com/Geohouse_Turku	Twitter	> 100 followers

B. The control question

"Next, we will present human-related statements. With these statements, we check that the respondents read the instructions carefully. We ask you to leave the options of Q12 blank and proceed to the next page.

Q12: What is your opinion on the following statements? On the scale 1–5, when 1 = totally disagree, and 5 = totally agree. We ask you to leave the options blank and proceed to the next page.

Most people try to apply the Golden Rule (do unto others as you would have them do unto you).
Every person has his or her own unique personality.
People are largely the masters of their own fate.
Most people would sneak into a movie without paying, if they could not get caught.
People usually tell the truth, even when they would be better off lying.
Our success in life is pretty much determined by forces outside our control.
Most people can control what happens in their lives.
Most of us would stop and help a person whose car is disabled at the side of the road.
People would act more responsibly if their living conditions improved."

The items were drawn from Philosophies of Human Nature subscales (Wrightsman 1974) as presented by Lupfer and Wald (1985). Some of the items were abbreviated.

C. The coding instructions

Below we introduce the coding instructions and the coding template. The examples introduced in the coding template are fictive but contain expressions from responses of the pilot study and the data applied in the analysis. The coders also had a reference table with example coding and notes on why the responses had been coded with the values in the table. (We do not present the table here, as it contains full responses from pilot participants). The instructions were translated from Finnish to English by the first author.

Instructions for coding

The analysis evaluates whether the responses fit the category criteria. This is estimated on the scale 0–2.
0 = the response does not fit the criteria of the category
1 = the response fits the criteria of the category, but respondent applies uncertain terminology
2 = the response fits the criteria of the category

We will mark the value "1" or "2" to the category cell if the response fits the criteria.

If the response does not fit the criteria of any category, we will add value ”1” to the category Ambiguous.

The responses that express annihilation after death are coded to at least one of the following: Annihilation body, Annihilation mind, Annihilation no distinction.

If the response is difficult to categorize, it is good to write this down in the coding diary. Diary notes are written in the outmost right column of the template during the coding process.

Code	Scale	Definition	Examples: What is coded in this category?	Examples: What is not coded in this category?
**Continuity social**	Value: 0–2	The respondent expresses that a human or some part of them exists after death socially (in other humans or community).	”In a way one could think that even after death we live on in the memories of our loved ones.” (1)<br><br>”Part of me continues in my children and grandchildren.” (2)	”In death we cease to exist.” (This response is coded merely to Annihilation no distinction.)<br><br>“At least I don’t believe in any life after death.” (The response is coded merely to Continuity rejection.)<br><br>”The thought of an afterlife feels comforting, but I just cannot believe it.” (The response goes to the categories Conflicted and Continuity comforting.)
**Continuity close others**		The respondent expresses that a human or some part of them exists after death in close others, e.g., in their memories.	”Part of us continues in the thoughts of our loved ones.” (2)<br><br>”In a way one could think that even after death we live on in the memories of our loved ones.” (1)	
**Continuity offspring**		The respondent expresses that a human or some part of them exists after death in their offspring.	”Part of me continues in my children and grandchildren.” (2)	
**Continuity societal**		The respondent expresses that a human or some part of them exists after death in their societal contribution, e.g., through work.	“My scientific work will be remembered even after I am gone.” (2)	
**Continuity mind/ consciousness**		The respondent expresses that a human’s mind/consciousness or some part of it exists after death.	”Our body decomposes but our mind continues.” (2)	
**Continuity soul**		The respondent expresses that a human’s soul or some part of it exists after death.	”The body decays but the soul moves on.” (2) [We also included ”spirit” in this category.]	
**Continuity other religious/New Age**		The respondent expresses that a human or some part of them exists after death and the respondent applies terminology that is often associated with religiosity or New Age.	”I believe we return to God, in Heaven.” (2)<br><br>“We continue [our existence] through reincarnation.” (2)<br><br>”I believe that God provides us with new bodies.” (2)	”The body decays, but the soul moves on.” (-> Continuity soul)
**Continuity same/new body**		The respondent expresses that a human or some part of them exists after death in some kind of a body.	“We continue [our existence] through reincarnation.” (2)<br><br>”I believe that God provides us with new bodies.” (2)	
**Continuity natural laws**		The respondent expresses that a human or some part of them exists after death and states this as a natural law or regularity, or associates this with a natural law, such as regularities of physics or biology.	”Our atoms return to the circulation of nature.” (2)<br><br>”Our body disintegrates. However, nothing disappears fully, see the law of conservation of energy.” (2)<br><br>”I cease to exist as a conscious individual, but someone could think that my genes continue.” (1)	"Each one of us dies. That’s how the nature works." (-> Annihilation no distinction)

**Continuity other**		The respondent expresses that a human or some part of them exists after death, but they do not specify their view or it does not fit any other continuity category.	”We continue somewhere else.” (2)<br><br>”We’ll be reunited in an afterlife.” (2)<br><br>"Death likely isn’t the end." (1)	”I believe we return to God, in Heaven.”<br>(-> Continuity other religious/New Age)<br><br>”The thought of an afterlife feels comforting, but I just cannot believe it.” (The respondent isn’t insecure of the view but does not believe in it and experiences conflict -> Conflicted and Continuity comfort.)
**Annihilation body/bodily functions**		The respondent expresses that the body or bodily functions disintegrate/end after death.	”Our body disintegrates. However, nothing disappears fully, see the law of conservation of energy.” (2)<br><br>”Our bodily functions cease and our consciousness ends.” (2)	
**Annihilation mind/ thoughts/ consciousness**		The respondent expresses that the mind annihilates after death.	”Our bodily functions cease and our consciousness ends.” (2)<br><br>"The mind ceases to exist." (2)<br><br>”Our body starts to disintegrate and our experiences cease.” (2)<br><br>”Our consciousness may be lost forever in death but we can’t know.” (1)	
**Annihilation no distinction**		The respondent expresses that a human or some part of them annihilates/ decomposes after death, but they do not separate the mind and the body.	“We cease to exist.” (2)<br><br>“Human flames out.” (2)	
**Annihilation life metaphor (e.g., sleep, prenatal state)**		The respondent describes annihilation after death by applying a metaphor that refers to biological] life.	”After death there’s nothing, it’s like an eternal sleep.” (2)<br><br>”Death is like returning to non-existence, the state before birth.” (2)	
**Science**	Value: 0–1	The respondent mentions a science-related term.	”Our body disintegrates. However, nothing disappears fully, see the law of conservation of energy.” (1)<br><br>”I cease to exist as a conscious individual, but someone could think that my genes continue.” (1)<br><br>”In accordance with current research, our existence is most likely limited, and at some point my consciousness likely ceases to exist.” (1)<br><br>”If science was more developed, human existence could be unlimited [in time].” (1)	”As the brain functions cease, the consciousness ends.”<br><br>“Our physical body rots.” [We decided to exclude ”physical” but include ”physiological”, as “physical” is a quite common expression in everyday Finnish when discussing the body.]

**Potential of science**		The respondent expresses that science/technology offers continuity as it develops.	”If science was more developed, human existence could be unlimited [in time].” (1)	
**Death good/ comforting/ beneficial**		The respondent expresses that there is something positive in death.	”We decompose and in this way make room for future generations.” (1)	
**Conflicted**		The respondent expresses that they experience conflicted feelings/thoughts over afterdeath. If the respondent does not deem the view they mention as plausible: the response is not coded as Continuity/Annihilation.	”The thought of an afterlife feels comforting, but I just cannot believe it.” (1)	”In death, our mind may be annihilated for good.” (The respondent does not express conflict.)
**Continuity comfort**		The respondent expresses that belief in continuity is or would be comforting (for themselves). If the respondent does not deem the view they mention as plausible: the response is not coded as Continuity/Annihilation.	”The thought of an afterlife feels comforting.” (1)<br><br>”The thought of an afterlife feels comforting, but I just cannot believe it.” (1)	
**Continuity rejection**		The respondent mentions continuity after death just to denounce it. The view is not coded as continuity.	”At least I do not believe in Heaven.” (1)	
**Ambiguous**		The response is difficult to interpret or it does not fit the criteria of any category. The response is coded only in this category.	"Get buried." (1)	

D. The study questionnaire (in Finnish)

<u>**Tutkimus maailmankuvista**</u>

Kutsu tutkimukseen

Kutsumme Sinut tutkimukseen, joka kartoittaa suomalaisten tiedettä arvostavia maailmankuvia. Tutkimus on osa Helsingin yliopistoon toteutettavaa väitöstutkimusta, jonka rahoittaa Suomen Kulttuurirahasto.

Kiitos siitä, että perehdyt vastaajille tarkoitettuun tietoon. Tämän sivun huolellinen lukeminen on tärkeää, jotta voit tehdä päätöksen osallistumisestasi.

Tietoa tutkimuksen osallistujalle

1. Tutkimuksen tarkoitus

Tutkimuksen tarkoitus on lisätä ymmärrystä tieteen merkityksestä suomalaisille. Tutkimus kartoittaa, millaisia maailmankuvia ja uskomuksia tieteen arvostamiseen liittyy.

2. Mitä minun tarvitsee tehdä?

Tutkimus koostuu yhdestä internetkyselystä. Kyselyn täyttäminen vie noin 30 minuuttia. Osallistuminen on täysin vapaaehtoista. Vaikka päättäisit osallistua tutkimukseen, voit keskeyttää vastaamisen milloin vain kertomatta syytä.

3. Kerätyt tiedot ja tietojen säilytys

Tutkimuksessa ei kerätä suoria tunnistetietoja, kuten nimiä. Käsittelemme tietoja EU:n yleisen tietosuoja-asetuksen (GDPR) mukaisesti. Aineisto säilytetään suojatulla levyasemalla. Aineistoa voidaan käyttää jatkotutkimukseen. Monivalintakysymyksillä kerätty anonyymi tieto voidaan tallentaa yhteiskunnalliseen tietoarkistoon tieteen avoimuuden edistämiseksi. Ainoastaan tutkimuksen toteuttavilla tutkijoilla on pääsy antamiisi avoimiin vastauksiin, ja ne tuhotaan viimeistään 1.1.2026.

4. Mitä hyötyä osallistumisestani on?

Osallistumisesi tutkimukseen lisää tietoamme nykysuomalaisten maailmankuvista. Erityisesti vastaukset avoimiin kysymyksiin ovat tärkeitä tämän kannalta. Vastausten avulla tutkimme, miten tieteen arvostaminen liittyy erilaisiin käsityksiin maailmasta.

5. Mitä tutkimustuloksille tapahtuu?

Vastauksesi koodataan ja yhdistetään muiden antamiin vastauksiin analyysia varten. Tulokset voidaan julkaista tutkimusartikkeleina sekä muina julkaisuina, ja niitä voidaan hyödyntää konferenssiesitelmissä. Tutkimuksen tietoja ei käytetä tavalla, joka mahdollistaisi yksittäisten vastaajien tunnistamisen.

6. Kuka on arvioinut tutkimuksen eettisyyden?

Helsingin yliopiston ihmistieteiden eettisen ennakkoarvioinnin toimikunta on antanut lausunnon tutkimuksen eettisyydestä. Tutkimus on suunniteltu yliopiston eettisten suositusten mukaisesti.

Arvostamme kutsun lukemiseen käyttämääsi aikaa. Halutessasi voit kysyä lisätietoja minulta sähköpostitse: [anonymized]. Tutkimuksen tietosuojaselosteen löydät [anonymized].

Parhain terveisin,

[anonymized]

Voit ottaa yhteyttä myös [anonymized].

Suostumus tutkimukseen

- Vahvistan, että olen lukenut tutkimuksen osallistujille tarkoitetun tiedon.
- Ymmärrän, että monivalintakysymyksillä kerätty anonyymi tieto voidaan tallentaa tietoarkistoon, jossa se on muiden tutkijoiden käytettävissä.
- Suostun osallistumaan tähän tutkimukseen.

Avoimet kysymykset

Seuraavaksi kysymme sinulta 1-2 kysymystä seuraavista teemoista: ihmisen alkuperä, kärsimys, elämän rajallisuus ja maailman alkuperä.

Olemme kiinnostuneita aiheisiin liittyvistä näkemyksistäsi. Kysymyksiin ei siis ole oikeita tai vääriä vastauksia. Kuulemme mielellämme myös pohdintaasi.

Käsitykset omasta alkuperästämme 1/2

K1: Suomalaisilla on useita käsityksiä siitä, miten ihmisen tietoisuus on syntynyt. Myös tiedeyhteisöissä kiistellään tästä kysymyksestä.

Millaisia käsityksiä sinulla on tietoisuutemme syntymisestä? (Yksilö- tai lajitasolla.)*

Käsitykset omasta alkuperästämme 2/2

K2: Tutkimusten mukaan ihmiset ja simpanssit polveutuvat yhteisistä esivanhemmista. Nämä esivanhemmat elivät vielä niinkin hiljattain kuin noin viisi miljoonaa vuotta sitten.

Pyydämme sinua kuvailemaan, miten ihmisellä ja simpanssilla voi olla yhteiset esivanhemmat.*

Käsitykset kärsimyksestä 1/2

Seuraavaksi pyydämme sinua kertomaan kärsimykseen liittyvistä näkemyksistäsi.

K3: Miksi pahoja asioita (esim. vakava sairaus) tapahtuu joillekin ihmisille enemmän kuin toisille?*

Käsitykset kärsimyksestä 2/2

K4: Miksi elämässämme mielestäsi on kärsimystä?*

Käsitykset kuolemasta 1/1

Seuraavaksi kysymme kuolemaan liittyvistä näkemyksistäsi.

K5: Mitä meille (ihmisille) mielestäsi tapahtuu kuoleman jälkeen?*

K6: Onko yksittäisen ihmisen olemassaolo mielestäsi ajallisesti rajallinen vai rajaton? (Voisitko kertoa meille, miksi ajattelet näin?)

Käsitykset maailmankaikkeuden alkuperästä 1/1

Ennen tieteeseen liittyviä monivalintakysymyksiä kysymme sinulta vielä maailmankaikkeuden alkuperästä.

K7: Nykykeskustelussa on esitetty useita näkemyksiä maailmankaikkeuden synnystä.

Miten näet maailmankaikkeuden saaneen alkunsa?*

Tiedekäsitykset 1/2

Pyydämme sinua arvioimaan tieteeseen liittyviä väitteitä.

K8a. Mitä mieltä olet seuraavista väittämistä? Asteikolla 1-5, kun 1 = täysin eri mieltä, ja 5 = täysin samaa mieltä.*

Tiede kertoo miten voimme tietää, mikä on totta.

Tiede lisää tarkoituksen ja merkityksen tunnetta elämässäni.

Tieteellinen tutkimus vahvistaa omaa identiteettiäni.

Tiede selittää maailman ilmiöitä.

Tiede antaa minulle kokemuksen, että elämäni on hyödytön.

Tiede antaa elämälleni suuntaa.

Tiede tekee elämästäni arvokkaamman.

Tiede motivoi minua.

Tiede vaikeuttaa oman elämäni ymmärtämistä.

Tiede vaikeuttaa oman olemassaoloni ymmärtämistä.

Tiede ja tutkimus lisäävät kokemustani siitä,
että elämälläni on merkitystä.

K8b. Mitä mieltä olet seuraavista väittämistä? Asteikolla 1-5, kun 1 = täysin eri mieltä, ja 5 = täysin samaa mieltä.*

Tieteellinen tutkimus on epäolennaista moraaliselle päätöksenteolle.

Arvioin ihmisten moraalista luonnetta tieteen avulla.

Tiede tarjoaa aineksia moraalisiin näkemyksiini.

Tiede tarjoaa minulle kehikon, jolla arvioida ihmiskunnan toimia.

Tiede tarjoaa minulle kehikon, jolla arvioida omia toimiani.

Tieteellinen tutkimus kertoo minulle, miten meidän tulisi toimia.

Tiede kertoo minulle, mikä on hyvää ja pahaa.

Tiede auttaa arvioimaan, onko ihmiskunta onnistunut vai epäonnistunut.

Tiede kertoo meille, mikä on arvokasta.

Tiedekäsitykset 2/2

Tällä sivulla pyydämme arvioimaan väitteitä, jotka koskevat tieteen merkitystä ihmiselle.

K9a. Mitä mieltä olet seuraavista väittämistä? Asteikolla 1-5, kun 1 = täysin eri mieltä, ja 5 = täysin samaa mieltä.*

Tieteellinen tutkimus vähentää toivoa ihmisen kuolemattomuudesta.

Tieteellinen tutkimus vähentää toivoa tietoisuuteni säilymisestä kuoleman jälkeen.

Tiede mahdollistaa elinikäni pitkittämisen.

Tiede antaa toivoa siitä, että läheiseni voivat välttää kuoleman.

Tiede antaa toivoa siitä, että voin itse välttää kuoleman.

Tieteellinen tutkimus tarjoaa tunteen omasta jatkuvuudestani kuoleman jälkeen.

Tiede mahdollistaa sen, että olemukseni säilyy kuolemani jälkeen.

K9b. Mitä mieltä olet seuraavista väittämistä? Asteikolla 1-5, kun 1 = täysin eri mieltä, ja 5 = täysin samaa mieltä.*

Koen, että tiede yhdistää minut sukupolvien ketjuun.

Koen, että tiede yhdistää minut osaksi eläinkuntaa.

Koen, että tieteellinen tutkimus kiinnittää minut ihmiskuntaan.

Koen, että tiede yhdistää minut luonnon kiertokulkuun.

Tiede lisää kokemustani olemassaolon rajallisuudesta.

Tiede lisää sen todennäköisyyttä, että minut muistetaan kuolemani jälkeen.

Tiede tarjoaa minulle kunnioittavan ihmettelyn kokemuksen (ns. ”awe-kokemuksen”).

Koen, että tiede erottaa minut ihmiskunnan muusta historiasta.

Tiede saa minut tuntemaan itseni osaksi maailmankaikkeutta.

Tiede saa minut tuntemaan itseni osaksi jotain muuta kokonaisuutta, mitä:

K10: Voisitko kertoa lyhyesti, millaista tiedettä ajattelit vastatessasi kysymyksiin?

Maailmankuvamittari ja taustatiedot

Pyydämme sinua vielä vastaamaan monivalintakysymyksiin maailmankuvastasi. Lisäksi kysymme sinulta muutaman yleisluontoisen taustatiedon.

Lopuksi voit osallistua arvontaan ja näet tutkimuksen vastaajille tarkoitetun tiedon.

Maailmankuvamittari 1/2

K11: Mitä mieltä olet seuraavista väittämistä? Asteikolla 1-5, kun 1 = täysin eri mieltä, ja 5 = täysin samaa mieltä.*

Maailma on oikeudenmukainen paikka.

Kaikella on tarkoituksensa.

Ihmisillä on pääosin hyvät tarkoitusperät.

Ihmiset saavat lopulta ansionsa mukaan.

Ihmisen olemus säilyy hänen kuolemansa jälkeen.

Elämäntapahtumat ovat satunnaisia siinä mielessä,
ettei niillä ole tarkoitusta.

Ihmisen mieli on jokseenkin erillinen kehon toiminnasta.

Ihmisellä on sielu.

Ihmisellä on vapaa tahto, joka ei riipu hänen kehostaan.

Riittävän kehittynyt koneäly voisi periaatteessa ajatella kuten ihminen.

Uskon kohtaloon.

Uskon kuolemanjälkeiseen elämään.

Tapahtumilla on Jumalan määräämä tarkoitus.

Seuraavaksi esitämme ihmisiin liittyviä väitteitä. Niillä tarkistamme, että vastaajat lukevat ohjeet huolellisesti. Pyydämme sinua jättämään kysymyksen K12 vaihtoehdot tyhjiksi ja siirtymään seuraavalle sivulle.

K12: Mitä mieltä olet seuraavista väittämistä? Asteikolla 1-5, kun 1 = täysin eri mieltä, ja 5 = täysin samaa mieltä. Pyydämme jättämään vaihtoehdot tyhjiksi ja siirtymään seuraavalle sivulle.

Suurin osa ihmisistä pyrkii noudattamaan kultaista sääntöä (tee kuten haluaisit itsellesi tehtävän).

Jokaisella ihmisellä on ainutlaatuinen persoonansa.

Ihmiset ovat pitkälti oman onnensa seppiä.

Enemmistö ihmisistä livahtaisi elokuvateatteriin maksamatta, jos ei jäisi siitä kiinni.

Ihmiset puhuvat usein totta silloinkin, kun se ei hyödytä.

Menestyksemme elämässä riippuu pitkälti tekijöistä, joihin emme voi vaikuttaa.

Enemmistö ihmisistä voi hallita sitä, mitä heidän elämässään tapahtuu.

Suurin osa meistä pysähtyisi auttamaan ihmistä, jonka auto on hajonnut tienposkeen.

Ihmiset käyttäytyisivät vastuullisemmin, jos heidän elinolosuhteensa kohenisivat.

Halutessasi voit kommentoida vastauksiasi tähän:

Maailmankuvamittari 2/2

Seuraavaksi pyydämme sinua arvioimaan luontoa koskevia väitteitä.

K13: Mitä mieltä olet seuraavista väittämistä? Asteikolla 1-5, kun 1 = täysin eri mieltä, ja 5 = täysin samaa mieltä.*

Luonto on elävä olento.

Jokin suurempi voima loi maapallon ja elävät olennot (eläimet, kasvit)
ja vaikuttaa näihin edelleen.

Jokin suurempi voima loi maapallon ja elävät olennot (eläimet, kasvit),
muttei enää vaikuta näihin.

Jumala loi maapallon ja elävät olennot (eläimet, kasvit).

Maapallon ja elävien olentojen (eläimet, kasvit) synty on pelkästään fysikaalisten tapahtumien ketju.

Ihminen on kehittynyt muista, varhaisemmista eläinlajeista.

Luonnonvalinnan seurauksena heikot yksilöt karsiutuvat populaatiosta.

Luonnonvalinnan tarkoitus on karsia heikot yksilöt populaatiosta.

Elämme todennäköisimmin jonkun toimijan (kuten koneälyn) luomassa todellisuudessa.

Luonto ylläpitää tasapainoa itsesäätelyllä.

Kaikki maailmassa koostuu materiasta tai palautuu materiaan/energiaan.

Eläinlajit voivat muuttaa biologisia ominaisuuksiaan selviytyäkseen.

Maailmassa on jokin tarkoituksellinen voima (esim. elämänvoima).

Kaikki ihmiset koostuvat samasta materiasta (esim. aine/energia, tähtipöly).

Kaikki ihmiset on tehty samasta materiaalista (esim. aine/energia, tähtipöly).

Halutessasi voit kommentoida vastauksiasi tähän:

Ennen taustatietoja kysymme sinulta vielä yhden tieteeseen liittyvän kysymyksen.

K14: Mitä mieltä olet seuraavista tieteeseen liittyvistä näkemyksistä? Asteikolla 1-6, kun 1 = täysin eri mieltä, 6 = täysin samaa mieltä.*

Tiede tarjoaa meille paremman ymmärryksen maailmankaikkeudesta kuin uskonto.

”Demonien riivaamassa maailmassa tiede on kynttilä pimeydessä.” (Carl Sagan)

Rationaalisesti voimme uskoa vain siihen, mikä on tieteellisesti todistettavissa.

Tiede kertoo kaiken tarvittavan siitä, mistä todellisuus koostuu.

Kaikki ihmisten kohtaamat haasteet voidaan ratkaista tieteellä.

Tieteellinen metodi on ainoa luotettava polku tietoon.

Tieteellinen tieto on ainoaa todellista tietoa, jota meillä voi olla.

Tiede on inhimillisen kulttuurin arvokkain osa.

Tiede on tehokkain keino saavuttaa totuus.

Tutkijoita ja tiedettä tulisi kunnioittaa enemmän nyky-yhteiskunnassa.

Voit halutessasi kommentoida vastauksiasi tähän:

Taustatiedot

Tämä on kyselyn viimeinen sivu. Toivomme sinun vastaavan taustatietoja kartoittaviin kysymyksiin, jotta voimme tarkastella näiden mahdollista yhteyttä vastauksiin.

K15: Mikä on sukupuolesi?*
- Nainen
- Mies
- Muu / En halua vastata

K16: Minkä ikäinen olet?*
- Alle 18 vuotta
- 18–30 vuotta
- 31–40 vuotta
- 41–50 vuotta
- 51–65 vuotta
- Yli 65 vuotta

K17: Kuinka monta vuotta olet opiskellut (alkaen ala-asteen ensimmäiseltä luokalta)?*

Opiskelulla tarkoitamme opintoja oppilaitoksissa.

K18: Kuinka henkisenä pidät itseäsi? 0 = ei yhtään henkisenä, 6 = erittäin henkisenä

Tässä käytetty henkisyyden käsite olisi englanniksi ’spiritual’.

K19: Kuulutko uskonnolliseen yhdyskuntaan?*
- Kyllä
- En

K20: Uskotko Jumalaan?*
- Kyllä
- En
- En osaa sanoa

K21: Mikä seuraavista kuvaa katsomustasi parhaiten?*
- Ateisti
- Agnostikko
- Ei uskontoa
- Välinpitämätön/neutraali
- Henkinen, muttei uskonnollinen
- Uskonnollinen
- Muu, mikä?

K22: Työskenteletkö tai oletko työskennellyt tutkimuslaitoksessa (esim. yliopistolla)?*
- Kyllä
- En

K23: Millä tutkimusalalla olet työskennellyt?*

[Vastaus oli 'Kyllä' kysymyksessä '36 [Tutkimustyo1]' (K22: Työskenteletkö tai oletko työskennellyt tutkimuslaitoksessa (esim. yliopistolla)?)]

- Humanistiset tieteet
- Yhteiskunta- ja sosiaalitieteet
- Matemaattiset tieteet ja luonnontieteet
- Muu

Halutessasi voit kommentoida vastauksiasi tähän:

[Vastaus oli 'Kyllä' kysymyksessä '36 [Tutkimustyo1]' (K22: Työskenteletkö tai oletko työskennellyt tutkimuslaitoksessa (esim. yliopistolla)?)]

Arvontaan osallistuminen ja palaute

Vastaajien kesken arvotaan yksi Amazon-lahjakortti (arvo 60 euroa). Toimitamme lahjakortin voittajalle sähköpostilla. Jos haluat osallistua arvontaan, pyydämme sinua lähettämään alla olevan tunnuksen sähköpostitse osoitteeseen: [anonymized]. Viestin otsikoksi voit merkitä: "Maailmankuvatutkimuksen arvonta".

Arvonnan tunnus: **4g678**

Arvostamme kyselystä antamaasi palautetta. Halutessasi voit kirjoittaa palautetta alla olevaan kenttään:

Voit siirtyä osallistujille tarkoitettuun tietoon painamalla "Lähetä".

[Osallistujien viimeinen sivu]

{if(Suostumus1_SQ001 == "" OR Suostumus1_SQ002 == "" OR Suostumus1_SQ003 == "", "Kiitos kiinnostuksestasi tutkimusta kohtaan. Valitettavasti tutkimukseen voivat osallistua vain ne, jotka 1) vahvistavat lukeneensa osallistujille tarkoitetun tiedon, 2) suostuvat anonyymin tiedon arkistoimiseen, ja 3) antavat suostumuksensa tutkimukseen.", "**Lämmin kiitos vastauksistasi!**")}

Tutkimuksen tarkoitus on lisätä ymmärrystä tieteen arvostamiseen liittyvistä käsityksistä. Olemme kiinnostuneita erityisesti uskonnollisista, uskonnottomista, edellä mainittuja yhdistelevistä näkemyksistä sekä usein yliluonnollisiksi kutsutuista käsityksistä.
Vastauksesi auttavat lisäämään ymmärrystämme suomalaisten maailmankuvien kirjosta. Lisäksi suomalaisilta saadut vastaukset auttavat monipuolistamaan kansainvälistä tutkimuskeskustelua. Keräämämme tiedon avulla tutkimme sitä, miten tieteen arvostaminen liittyy erilaisiin käsityksiin maailmasta.

Voit halutessasi saada tutkimusraportin tuloksista sähköpostiisi, kun tulokset julkaistaan. Jos haluat raportin, pyydän sinua lähettämään tiedon tästä sähköpostitse osoitteeseen: [anonymized].

Vastaan mielelläni kaikkiin tutkimusta koskeviin kysymyksiin.

Parhain terveisin,

[anonymized]

Tutkimuksen tietosuojaselosteen löydät [anonymized].

***

Pyydämme nyt sulkemaan selaimesi. Huom! Mikäli täytit kyselyn yhteiskäytössä olevalla tietokoneella (kirjastot, oppilaitokset, nettikahvilat jne.) tyhjennä selaimen välimuisti ja sivuhistoria.

Bibliography

Lupfer, Michael, & Kenneth Wald
1985 An Exploration of Adults' Religious Orientations and Their Philosophies of Human Nature. – *Journal for the Scientific Study of Religion* 24 (3), 293–304.
